# Mutation Breeding of *Monascus* to Produce a High Yield of Orange Pigment and Low Citrinin Content Using the ARTP Method

**DOI:** 10.3390/jof10080553

**Published:** 2024-08-05

**Authors:** Chan Zhang, Qing Sun, Le Yang, Arzugul Ablimit, Huijun Dong, Haijiao Wang, Congcong Wang, Chengtao Wang

**Affiliations:** 1 School of Food and Health, Beijing Technology & Business University (BTBU), Beijing 100048, China; 2 Beijing Advanced Innovation Center for Food Nutrition and Human Health, Beijing Technology & Business University (BTBU), Beijing 100048, China; 3 Beijing Engineering and Technology Research Center of Food Additives, Beijing Technology & Business University (BTBU), Beijing 100048, China

**Keywords:** *Monascus*, ARTP, orange pigment, citrinin, ultrasonic extraction

## Abstract

*Monascus* is a filamentous fungus with a long history of application in China, which can produce a variety of secondary metabolites, including Monascus red pigments, *Monascus* orange pigments, *Monascus* yellow pigments, and citrinin. There is widespread attention being paid to natural pigments because of their safety. Among the many natural pigments, orange pigment has a wide range of applications because of its unique color, but current production levels in the orange pigment industry are limited to a certain extent due to the insufficiently wide range of sources and low production. In this study, the ARTP mutation was used to obtain a strain with high-yield orange pigment and low citrinin. The strain RS7 was obtained through two-step mutagenesis, and all three pigments were improved to different degrees. The color value of orange pigment was elevated from the original 108 U/mL to 180 U/mL, an increase of 66.7% compared to the original strain, and the citrinin content was reduced by 69%. The result of microscopic morphology showed that RS7 has more wrinkles and is more convex than the R1 strain, but there was little change between the two strains. Therefore, the ARTP mutation influenced the growth and the biosynthesis of pigments in *Monascus*. In addition, the conditions of ultrasonic extraction of *Monascus* pigments were optimized using the response surface, and the separation of pigments was achieved with the method of thin-layer chromatography. Pigment stability results showed that the temperature had no significant effect on orange pigment, while tea polyphenol could improve its stability. This study generated a strain with high-yielding orange pigment and could lay a foundation for the future application of *Monascus* orange pigment in the food industry.

## 1. Introduction

As an important element in food, pigments play a decisive role in the sensory quality of food. And natural pigments are becoming increasingly popular as far as environmental impact and safety are concerned [1]. In addition, while they impart color to food, they also have physiological activities that are beneficial to human health [2]. These advantages make them popular natural compounds, and they are widely used in food, cosmetics, and the textile industry. *Monascus* pigments (MPs) have good safety, light resistance [3], heat resistance [4], and chemical resistance and are widely used in making vinegar, red fermented bean curd, marinated meat, red sausage, candy, jam, fruit drinks, pastries, and other foods requiring coloring. When using MPs instead of nitrite for hair color, the color effect is good and stable [5]. Therefore, microbial pigments have a great potential for application.

As a filamentous saprophytic fungus, *Monascus* is usually used to ferment rice to produce red mold rice, which has been used in traditional fermented food for thousands of years [6]. *Monascus* is aerobic and acid-resistant, with an optimal growth pH range of 3.5–5.0 and an optimal growth temperature range of 25 °C–30 °C. Most of the colonies are characterized by velvety hairy and radial patterns; the colonies are white at the beginning of growth, and the color changes from white to red and finally to dark red as the growth proceeds [7]. MPs are one of the major secondary metabolites produced during the fermentation of *Monascus* [8]. They are considered to be a kind of classic microbial pigment because of their better stability than other natural pigments. In addition, MPs have antibacterial activity against certain pathogenic microorganisms and can also reduce blood lipids and prevent obesity [9,10]. MPs are a series of polyketone mixtures, mainly including yellow pigments, orange pigments, and red pigments [11]. In addition, orange pigment in *Monascus* is firstly synthesized by acetyl CoA and malonyl CoA through a complex enzymatic reaction, which then produces red and yellow pigments, respectively. Orange pigment has a wide range of application scenarios because of its unique color among many MPs. The sources of natural orange pigments are mainly β-carotene [12] and *Monascus* orange pigment [13], but since β-carotene is mostly derived from plants, its production is greatly affected by seasons and regions, with unstable yield and high production costs, while *Monascus* orange pigment, as a natural pigment produced by the fermentation of *Monascus*, can be produced at a large scale all year round, with a high conversion rate and low cost.

However, citrinin, a mycotoxin, is also produced along with MPs. Hajjaj et al. [14] explored the synthetic pathway of ^13^C in part of tangerine using the isotopic labeling method, and the results showed that both citrinin and MPs were produced by the polyketide biosynthetic pathway. The first part of the pathway consists of one molecule of acetyl CoA and three molecules of malonyl CoA, which form a tetrone body catalyzed by the PKS enzyme. The pathway is then divided into two pathways, one of which is condensed with acetyl CoA to form citrinin, and the other is a polyketide chain with four keto groups condensed with malonyl CoA to form MPs. However, the regulation of the synthesis of citrinin is still unclear. At present, many countries have made requirements for minimum detection limits of citrinin [15]; Japan took the lead in 1999 to formulate the national control standard of citrinin in MPs, i.e., 0.2 mg/kg. Furthermore, other countries are also formulating relevant policies, and the content of citrinin in *Monascus* products can be produced and sold only under the limit. Due to the presence of citrinin, the application of *monascus* products is limited. How to improve the yield of MPs while reducing the content of citrinin has become one of the most urgent problems to be solved. Currently, there are some approaches to investigate and optimize production [16]. Firstly, the optimization of the medium composition can achieve the desired effect. Wang et al. explored the effects of some flavonoids on citrinin and pigments [17]. Zhu et al. showed that under selenium-enriched conditions, the biosynthesis of orange and red MPs was significantly enhanced [18]. Secondly, genetic engineering has been used recently to knock out or overexpress key genes to regulate the production of pigment and citrinin [19]. Moreover, the production of a high-yield pigment or a low-yield citrinin strain can be induced [20]. Methods of selecting high-yielding strains are more efficient than changing media conditions, with a high degree of enhancement and environmental sustainability, but traditional mutations are, to some extent, randomized.

Mutations are changes in the sequence of genes or proteins that lead to variation; some of these variations may be accompanied by changes in observable or measurable traits [21]. Mutation plays an important role in exploring gene function and is extensively used in genomics research [22,23]. Owing to the advantages of a low temperature and high energy, atmospheric-pressure room-temperature plasma (ARTP) is becoming a new type of mutagenesis tool [24]. The electrons and free radicals in the jet can directly damage the cell wall, change the permeability of the cell membrane, and promote the entry of various mutagens into the cell to act on the DNA and achieve the required mutation [25]. Compared to traditional mutagenesis methods, ARTP has multiple mechanisms for causing damage to genetic material, which can increase the diversity of mutants [26]. For example, ARTP mutagenesis was found to be effective in reducing acetaldehyde content, increasing tyrosine content, and improving the flavor stability of beer during fermentation; insights from this study suggested that ARTP mutagenesis may be achieved by altering the permeability of the cell membrane of *Saccharomyces cerevisiae* [27].

At present, there are many kinds of pigment-extraction methods, including solvent extraction and distillation extraction. These methods have low efficiency and low environmental protection [28]. Compared to traditional extraction methods, the cavitation effect and mechanical action produced by ultrasonic waves in ultrasonic extraction can rupture cells in a short time, facilitating the penetration of the solvent into the matrix and creating conditions for the release and dissolution of active ingredients [1]. Additionally, the effect of heat results in an increase in temperature, accelerating the diffusion of effective ingredients [29]. The ultrasound extraction technique has been applied widely to obtain compounds due to its ability to achieve high yields in less time [30,31]. Moreover, rather than using high-performance liquid chromatography (HPLC) methods, thin-layer chromatography may be used to separate orange pigment quickly and efficiently [32].

Therefore, this study aimed to generate a high-yielding orange pigment strain using ARTP. The ultrasonic extraction method supplemented with thin-layer chromatography was used to achieve the separation of MPs. Properties of the mutant strain were investigated; this will provide new scientific evidence that may be used to improve the production of *Monascus* orange pigment.

## 2. Materials and Methods

### 2.1. Strains and Materials

*Monascus purpureus R1*, strain number CGMCC 12,502, was obtained from the China General Microbial Culture Center and preserved by the Microbiology Laboratory of Beijing Technology and Business University.

*Monascus purpureus R1* and its mutant variants were cultured in a liquid medium containing 40 g/L of Indica rice flour, 8 g/L of peptone, 5 g/L of soybean meal, 2 g/L of KH_2_PO_4_, 2 g/L of NaNO_3_, and 1 g/L of MgSO_4_·7H_2_O. The planar spores were washed with 10 mL of sterile water, filtered through a double nylon cloth, and shaken well. We ensured that the number of spores in the same inoculum volume was approximately the same (about 10^5^) after counting with a hemocytometer plate.

Afterward, 5 mL of liquid was transferred to 50 mL of fermentation broth containing 77 g/L of Indica rice flour, 75 g/L of glucose, 2 g/L of soybean meal, 0.5 g/L of KH_2_PO_4_, 1.8 g/L of NaNO_3_, 1 g/L of MgSO_4_·7H_2_O, and 3.5 g/L of corn steep liquor.

Potato Dextrose Agar (PDA), glucose, peptone, soybean meal, MgSO_4_·7H_2_O, KH_2_PO_4_, NaNO_3_, corn steep liquor, filter paper, ethanol, glutaraldehyde, phosphate-buffered saline (PBS), isoamyl acetate, hexamethyldisilazane, H_2_O_2_, tea polyphenol, toluene, and ethyl acetate were purchased from Beijing Aoboxing Bio-technology Limited Liability Company (Beijing, China).

### 2.2. Preparation of Different Spore Concentrations

The *Monascus R1* strains were cultured on the PDA medium for 5 days, and the spores in the strains were transferred to the sterile saline until the sterile saline became dark red. Then, 100 µL aliquots of the saline were taken, subjected to different dilution multiples, and spread evenly on a PDA tablet. The total number of colonies was expressed as colony forming unit (CFU) [33].

### 2.3. ARTP Mutagenesis and Mutagenic Lethality

A special metal carrier was soaked in 70% (*v*/*v*) ethanol, exposed to an alcohol lamp burning cool ARTP (Beijing Siqing-yuan Biological Technology Limited company, Beijing, China), and placed into a one-time flat dish (NEST Petri Dish 90 mm, Li Hui Medical Instrument Limited Company, Taizhou, China). Then, 10 µL *Monascus* R1 liquid with a concentration of 10^6^–10^7^ CFU/mL was placed evenly on the metal carrier. The metal carrier was placed in 2 mL centrifugal tubes containing 1 mL of sterile saline and then placed in the oscillator instrument (Vortex-Qilinbeier5; The Forest Belle Instrument Manufacturing Limited Company, Haimen, China) for 5 min. It was then cultured on the PDA for 5 d.

Viable counts were determined at 30, 40, 50, 60, 70, 80, 90, 100, 110, and 120 s to calculate the lethal rate of ARTP treatment, and the lethal curve was obtained. The lethal rate (%) was calculated as ([U − T]/T) × 100, where U was the number of colonies without ARTP treatment (CFU/mL), and T was the number of colonies after ARTP treatment (CFU/mL).

### 2.4. Screening of Strains

The selected ARTP mutants of *Monascus,* whose colonies were orange on the dishes, were proliferated through sub-cultivation on new dishes with PDA, respectively. Thereafter, the mutants were grown in seed solution for 2 days before being transferred to the fermentation broth for further proliferation for 12 days. At the end of the cultivation, the orange color values of the fermentation liquid were detected as described below, and the one with the highest value was selected for a further round of screening operations from ARTP to detect orange color values. The strains with the highest productivity of orange pigment were ultimately selected for the following test.

### 2.5. Determination of Pigments Color Value and the Comparison of Colony Color

A total of 3 mL of fermentation culture of the original strain and mutant strains was collected after 2, 5, 8, 12, and 15 days for detection (adjusted to a concentration of 10^6^ spores/mL). The fermentation culture with 6 mL of 70% ethanol was placed in the water bath at 60 °C for 1 h, followed by centrifugation for 15 min at 4000 rpm/min. The supernatant after dilution was measured using a spectrophotometer (UV-2450, Shimadzu, Corporation, Kyoto, Japan). Although strain RS7 underwent a mutagenic process, the physicochemical properties of the pigments were consistent with those of strain R1. The absorbance values of red, orange, and yellow pigment in these two strains were measured at the same wavelength, 505, 465, and 410 nm, respectively, which possess characteristic absorption peaks. *Monascus* color value = absorbance value × dilution factor.

The suspension prepared from the original and rescreened strains were separately incubated on a PDA medium for 5 days to observe the effect of mutation on the color of the *Monascus* colonies.

### 2.6. Determination of Citrinin Content

A volume of fermented liquid of the original strain and mutant strain (adjusted to a concentration of 10^6^ spores/mL) was added to twice its volume of 70% methanol and homogenized at high speed for 2 min, then centrifuged at 3000 rpm/min for 15 min. A total of 1 mL of the filtrate was diluted and mixed with 39 mL of 10 mmol/L phosphoric acid solution (pH 7.5). This was then filtered through a 0.22 µm filter, purified using an immunoaffinity column, and measured with HPLC (HPLC; Agilent, Santa Clara, CA, USA).

A C18 column (5 µm, 25 cm × 4.6 mm) was used for analytical separation. Acetonitrile as mobile phase A and 0.1% phosphoric acid as mobile phase B were chosen for gradient elution detection with a flow rate of 0.7 mL/min. A fluorescence detector was used for the measurement, with the excitation wavelength at 350 nm, emission wavelength at 500 nm, and an injection volume of 10 μL. A citrinin standard (Shanghai Yuanye Bio-Technology Co., Ltd., Shanghai, China) was used to construct a standard curve with five points over a range of 5–100 mg/mL.

### 2.7. Determination of Dry Weight of Mycelium

The fermentation broth of the original strain and the mutant strains after 2, 5, 8, 12, and 15 days were collected for detection. A 5 mL fermentation broth was filtered (adjusted to a concentration of 10^6^ spores/mL) with four layers of gauze that had been weighed with analytical balance (Sartorius BSA224S, Saiduolisi Scientific Instrument Limited Company, Beijing, China) and washed with sterile water until the filtrate was colorless. After that, the gauze with the mycelium was put in the oven (FCD-3000 Serials; Friwide Automation Equipment Limited Company, Shanghai, China) to dry to constant weight at 60 °C. The gauze was weighed again after drying, and the difference between the two weights was the dry weight of the strains.

### 2.8. Observation of Scanning Electron Microscope (SEM)

*Monascus* R1 and its mutant strains on the 8th day of fermentation were collected and centrifuged for 5 min at 12,000 rpm/min and then resuspended in 2.5% glutaraldehyde solution for 12 h. The cells were washed twice with 0.1 M of PBS, and the supernatants were discarded. The cells were dehydrated twice using different concentrations of ethanol (30, 50, 70, 80, 90, and 100%). Each concentration was washed for 10 min, followed by centrifugation for 5 min at 4 °C at 12,000 r/min, and the supernatants were discarded. The ethanol was replaced with isoamyl acetate and ethanol (*v*:*v* = 1:1) and an isopentyl acetate solution in that order; the previous operation was then repeated. Hexamethyldisilazane was added in the end, and the centrifuge tubes were plugged with absorbent cotton and put in the oven at 60 °C until the sample was dried [34]. The sample was observed using scanning electron microscopy (SU8010; Hitachi, Tokyo, Japan).

### 2.9. Optimization of Ultrasonic Extraction Conditions for Pigments

Cavitation bubbles collapse to the solid surface. The micro jet released by bubbles causes the surface to peel off and particle fragmentation. The impact force can destroy the cell wall and accelerate the release of intracellular compounds that dissolve in the solvent. In order to improve the extraction of pigments, single-factor experiments, including ethanol concentration, liquid-to-material ratio, and ultrasonic time, were carried out, combined with the response surface method to optimize the extraction conditions [35].

Ethanol was chosen as the extraction medium, and different concentrations of ethanol solutions, including 30%, 40%, 50%, 60%, 70%, 80%, and 90%, were prepared for extraction. The liquid-to-material ratio refers to the ratio of the volume of the extracted solution to the weight of the extract, which has an influence on the extraction result. An experiment range of 10–70 mL/g with a total of seven levels (10, 20, 30, 40, 50, 60, and 70) was used for exploration. The setting of the single-factor experiment time included seven levels and was consistent with the liquid-to-material ratio.

### 2.10. Pigment Separation of Mutant Strain

The optimal ultrasonic pigment extraction conditions on the vacuum rotary evaporator (IKA HB 10 control; Eika Equipment Limited Company, Guangzhou, China) were as follows: 40 °C, 20 KPa spin steaming, rotary steam volume of approximately 30% of the original volume, and capillary (Glass Sample Capillary; Instrument Factory of West Chinese Medical University, Hangzhou, China) at a 2 cm distance from silica gel (Silica Gel GF254 Plate; Qingdao Ocean Chemical Limited Company, Qingdao, China). The unfolding agent was a mixture containing toluene:ethyl acetate:formic acid (V:V:V) at a ratio of 7:3:1). A total of 40 mL of expansion agent was added in the chromatographic cylinder (Double Groove TLC Cylinder P1-2020; Shanghai Chu Analysis Instrument Limited Company, Shanghai, China); the chromatography duration was 30 min to achieve the separation of MPs. Following that, the silica gel plate was put in the oven and baked for 5 min at 40 °C. The orange part was scraped off from the silicone board with a knife and then dissolved in the ethanol reagent that had been filtered with a 0.45 µm nylon filter (MREDA, Wallace Ruida Science and Technology Limited Company, Beijing, China). Samples were prepared for stability testing.

### 2.11. Stability of Orange Pigment of Mutant Strains

The pigment samples obtained from the separation were divided into three groups. All operations were kept away from light, and the control group was stored at 25 °C without any treatment. In experimental group 1, the absorbance value of each orange pigment was measured at 40, 50, 60, 70, 80, 90, and 100 °C for 8 h. Compared to the control group, the absorbance value of the orange pigment in experimental group 2 with the addition of tea polyphenols (controlled at a concentration of 1 g/L) and fermentation at 25 °C was measured after 1, 2, 3, 4, 5, 6 and 7 days, respectively.

### 2.12. Statistical Analysis

All experiments were performed independently in triplicate. Statistical analysis was performed using SPSS Statistics 24 software. A one-way analysis of variance (ANOVA) was used to determine the significance of the differences between the control and experimental groups.

## 3. Results

### 3.1. The Lethal Rate of Mutant Strain and Screening of Strain

**Figure 1:**
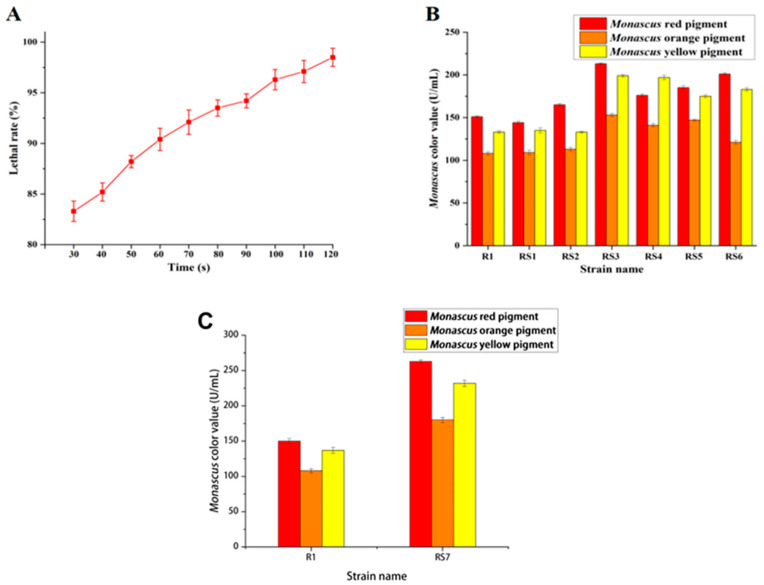
Effect of mutation time on the lethal rate of *Monascus* R1 (**A**); comparison of pigment color values for *Monascus* R1 and six strains obtained through primary screening (**B**); comparison of pigment color values for *Monascus* R1, RS7, and two strains obtained through secondary screening (**C**).

Preliminary experiments showed that ARTP had a high lethal rate for *Monascus* R1, and the lethality curve of *Monascus* R1 was determined with the PDA medium viable count method. Figure 1A shows that when the mutation time was 30 s, the lethal rate reached 82.3%; it increased to 98.5% at 120 s. Shorter mutation times changed the color slightly; however, the longer the time, the higher the lethal rate, which led to no colony growth on the PDA medium. Therefore, 70 s, 80 s, and 90 s were chosen for mutation in this experiment.

Through ARTP mutagenesis, six mutants were obtained that showed orange pigment production and were named *Monascus* RS1, RS2, RS3, RS4, RS5, and RS6. Then, the color value of the strains was measured on day 12, the time point of the highest pigment production during the fermentation (Figure 1B). Among them, the change of strain R3 was relatively obvious, with the color values of red, orange, and yellow pigment raised by 41%, 49.6%, and 41.6%, respectively. After the initial screening, a secondary mutation was adopted for strain RS3 to ensure the maximization of the mutation effect in this experiment; the most effective mutant strain was obtained and named RS7 (Figure 1C).

### 3.2. Pigment Biosynthesis and Cell Growth

**Figure 2:**
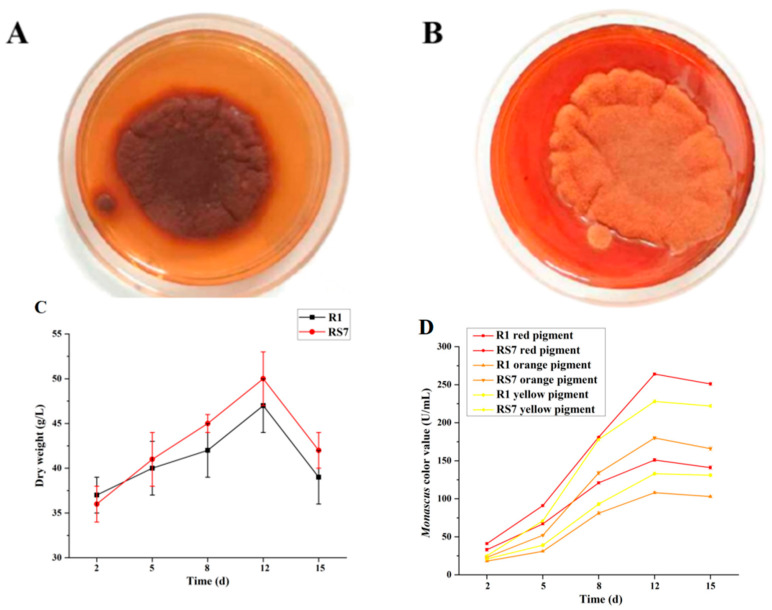
Effect of mutagenesis on the colony color grown on the PDA medium of *Monascus* R1 (**A**) and *Monascus* RS7 (**B**); effect of mutagenesis on the dry weight (**C**) of strain *Monascus* R1 and strain *Monascus* RS7; pigment color values contrast between *Monascus* R1 and *Monascus* RS7 (**D**).

Then, the color values of *Monascus* R1 and *Monascus* RS7 were measured at key points during the 15-day fermentation cycle (Figure 2D). The trends for the three pigments were generally consistent, and the color value showed an upward trend in the early stage until it reached its peak on the 12th day, followed by a decline. The color value of the red pigment reached 263 U/mL, an increase of 75.1% from R1, and the color value of the yellow pigment reached 220 U/mL, an increase of 71.4% from R1. Similarly, on the 12th day, the color value of the orange pigment reached its maximum improvement: it increased by 66.7% from 108 U/mL to 180 U/mL. On the 15th day of fermentation, the experimental results obtained through the HPLC method were analyzed, and it was found that the citrinin content of *Monascus* RS7 decreased compared to that in *Monascus* R1, from 0.53 mg/L to 0.16 mg/L, a decrease of 69%. Additionally, the color change of *Monascus* R1 and *Monascus* RS7 colony cultured for 5 days on PDA medium was observed. *Monascus* R1 (Figure 2A) was dark red, while the mutant strain *Monascus* RS7 (Figure 2B) showed a bright orange color, and the medium was pale red. *Monascus* RS7 retained the main traits of *Monascus*. Macroscopically, its epidermal folds formed pigments and secreted them into the medium to color it; more orange pigment was secreted (and observable with the naked eye) compared to that secreted by *Monascus* R1.

Biomass can indicate the growth of different *Monascus* strains. The findings showed no significant difference between the dry weights of RS7 and R1, except an increase from 47 to 50 g/L (6.38%) (Figure 2C). The mutations had little effect on the growth of the strains and did not alter genes associated with basal growth to any great extent.

**Figure 3:**
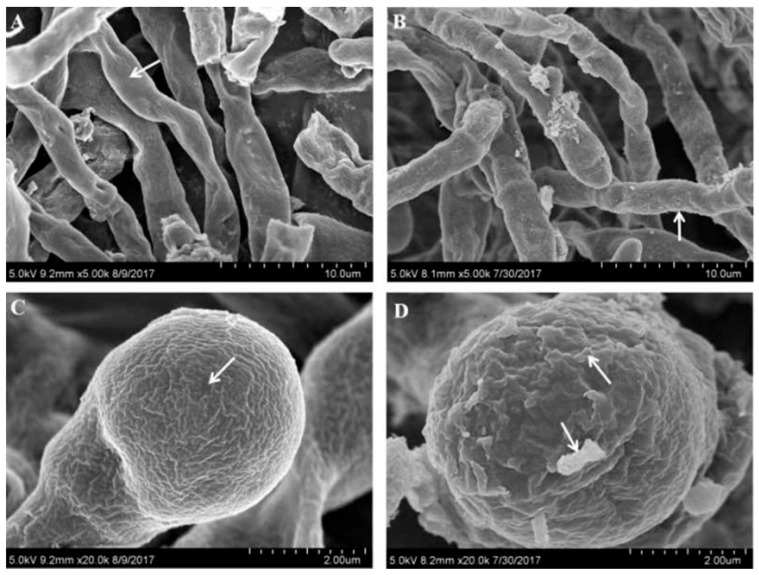
Scanning electron micrographs of *Monascus* R1 (**A**,**C**) and *Monascus* RS7 (**B**,**D**) after eight days of cultivation. Results were taken at different magnifications: (**A**,**B**) 5000 magnification, (**C**,**D**) 10,000 magnification. Arrows indicate folds.

The fermentation broth on the 8th day of *Monascus* R1 (Figure 3A,C) and *Monascus* RS7 (Figure 3B,D) was processed and observed with SEM. Compared to RS7 (Figure 3B,D), R1 (Figure 3A,C) had a smoother surface with better particle fullness, and the spore surface folds were shallow. However, there were very fine filamentous substances on the surface of the mutant strain RS7, and the degree of mycelium wrinkle was greater than that of R1.

### 3.3. Single-Factor Extraction and Response Surface Optimization for Ultrasonic Extraction

After obtaining a mutant strain with a high production of orange pigment, we extracted it and discussed the effects of ethanol concentration, feed-liquid ratio, and ultrasonic time on the extraction of the pigment.

**Figure 4:**
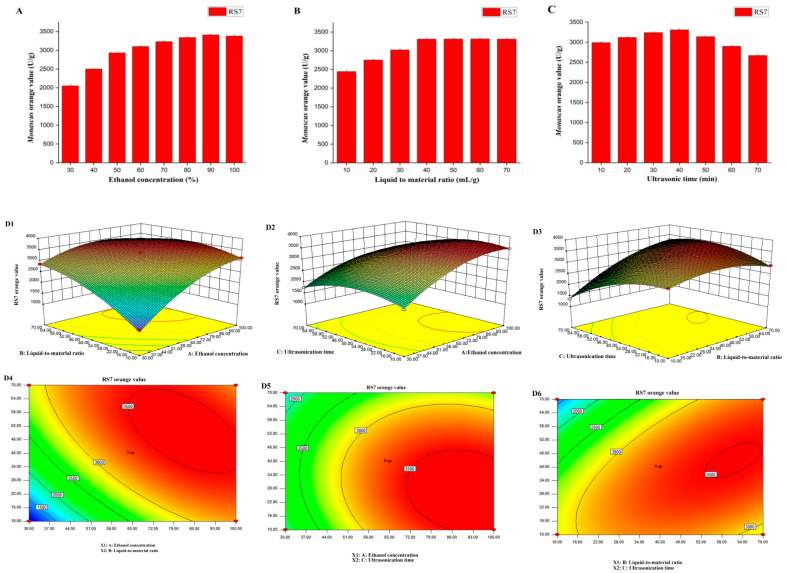
Single-factor experiment based on ultrasonication extraction, ethanol (**A**), liquid-to-material ratio (**B**), and ultrasonication time (**C**) were chosen as single factors. Three-factor response surface experiments using ethanol, liquid-to-material ratio, and ultrasonication time (**D1**–**D6**) as variables. Three-dimensional diagram of ethanol concentration, liquid-to-material ratio, and response surface (**D1**); three-dimensional diagram of ethanol concentration, ultrasonication time, and response surface (**D2**); three-dimensional diagram of liquid-to-material ratio, ultrasonication time, and response surface (**D3**); three-dimensional projection of response surface, ethanol concentration, and liquid-to-material ratio (**D4**); three-dimensional projection of response surface, ethanol concentration, and ultrasonication time (**D5**); and three-dimensional projection of response surface, liquid-to-material ratio, and ultrasonication time (**D6**).

Figure 4A shows that with the increase in the ethanol concentration, the color of the orange pigment also increased before the ethanol concentration reached 90%. Based on the principle of similar compatibility, the fact that orange pigment is organic and dissolves more easily in ethanol than in water could explain the phenomenon. However, after the dissolution reached saturation, even if the concentration continued to increase, the color value did not change much.

Figure 4B shows that the color value gradually improved with the increase in the liquid-to-material ratio until it reached 40. Afterward, the pigment value remained unchanged. Under the condition that the weight of the extracted material was certain, a lower liquid-to-material ratio meant that too little extraction medium may not cause sufficient dissolution of the pigment.

Figure 4C shows that with the increase in the ultrasonic time, the pigment value increased slowly. After reaching the highest value at 40 min, it began to show a downward trend. The longer time resulted in a stronger thermal effect produced by ultrasound, which negatively impacted the stability of the pigment.

According to the response surface experiment (Figure 4D1–D6), when the concentration of ethanol was 81.46%, the ratio of liquid-to-material was 39.42, the ultrasonic time was 43.58 min, and the highest orange value of *Monascus* was 3083.92 U/g. We selected an ethanol concentration of 81%, ultrasonic time of 44 min, and liquid-to-material ratio of 39 for verification, and the result of the *Monascus* orange value was 3010 U/g with an error of 2.39%.

### 3.4. MPS Separation

**Figure 5:**
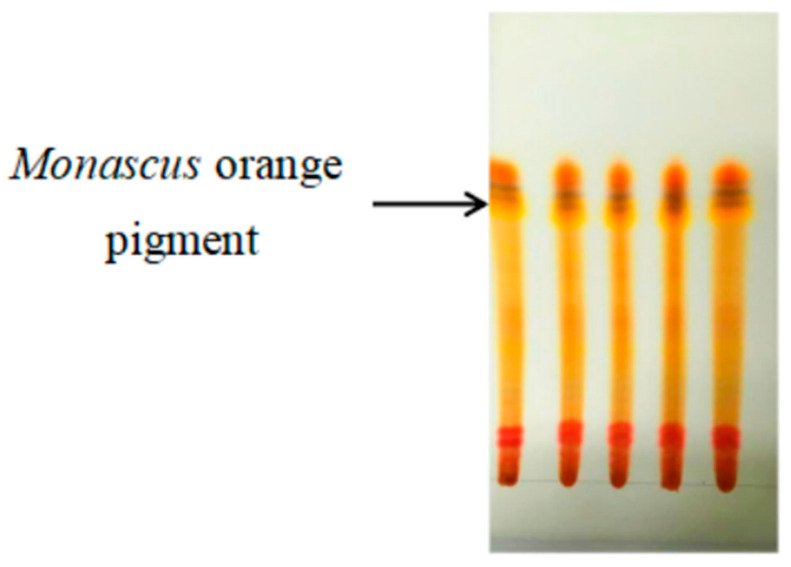
Separation of MPs on the GF254 silica gel plate.

Pigments are generally separated using a column and a silica gel plate [36,37]. The latter was chosen in this research. Preliminary experiments showed that when toluene, ethyl acetate, and formic acid were selected as the expansion agent (V:V:V = 7:3:1), the separation effect was greater, and primary separation of the orange pigment from the red pigment was achieved (Figure 5).

### 3.5. Stability of MPs

**Figure 6:**
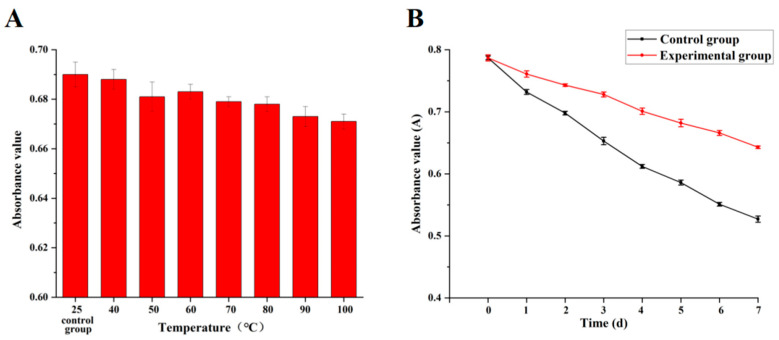
Effect of the temperature (**A**) and the addition of tea polyphenols (**B**) on the stability of orange pigment of strain RS7.

Figure 6A shows that temperature had little effect on *Monascus* orange pigment; this is consistent with the research conducted by Zheng and Yao [38]. Additionally, tea polyphenols, as an antioxidant, can be used as a stabilizer to improve the situation. Therefore, the mutant strains were chosen in this experiment to explore the effects of the presence of tea polyphenols on the stability of orange pigment. The findings showed that it could improve the stability of the pigment; its presence protected the pigment to a certain extent and reduced the degradation (Figure 6B). Since the stability of the natural pigment was poor, as the test days continued, the natural degradation of pigments could not be avoided, even in the presence of tea polyphenols.

## 4. Discussion

Microorganisms undergo a series of chemical and physical changes after various mutagenic treatments, which will be expressed in the subsequent growth process. At present, ARTP mutagenesis technology has been maturely utilized in microbial breeding and has made some progress in crop breeding [39,40]. Due to the difficulty in obtaining high-yield pigment and low citrinin-producing mutants through one-step mutation, two-step mutagenesis was chosen to achieve the desired effect [41]. At the end of the first mutagenesis step, we selected the strain with the highest color value according to the yield of orange pigment for the second mutagenesis. This strain was named RS7, and the yields of red pigment, orange pigment, and yellow pigment were improved compared to the original strain, with increases of 75.1%, 66.7%, and 71.4%, respectively.

It is worth mentioning that the synthesis mechanism of MPs is complex. First, the orange pigment is synthesized, then the yellow pigment is produced through reduction reactions, and the red pigment is produced through amination [42]. Therefore, we hypothesized that once the synthetic yield of orange pigment increased, the other two pigments would show the same trend subsequently. Notably, the citrinin content of *Monascus* RS7 was reduced. The synthesis pathway of citrinin is consistent with that of MPs in the first stage; however, in the second stage, citrinin was synthesized alone. From this, we hypothesized that mutagenesis may have caused the expression of the relevant synthetic enzymes in the second phase to be inhibited, but this still needs to be further evaluated. A correlation between mycelial morphology and secondary metabolites in filamentous fungi was reported [43]. According to the results, the mutation, to a certain extent, did not cause a big change in the mycelium dry weight. Compared to the starting strain, after mutagenesis, the mycelium dry weight only increased slightly, and the difference was not significant. In the electron microscopic results, compared to the starting strain, due to the mutation of filament smoothness and wrinkle degree, certain differences were observed, leading to the change in the morphology of *Monascus*; this may be a reason for the change in pigment yield. Chen et al. [44] found that the high-salt stress fermentation had an impact on the yellow pigment-related genes and yield and described the importance of mycelial morphology to pigment biosynthesis.

In recent years, significant efforts have been made by researchers to improve the yield of pigments, with the focus being on optimizing the culture medium and fermentation conditions, as well as genetic modification at the molecular level with genetic engineering techniques. Patrovsky et al. [45] found that the addition of 8.8 g/L of peptone and an initial pH of 2.5 can effectively promote the production of yellow and orange pigments. Liu et al. [46] demonstrated that lanthanum (III) ion (LaCl3) could induce red pigment biosynthesis and possesses the potential to inhibit the production of citrinin. Liu et al. [47] constructed a mrPDE-gene-knockout mutant and found that the cAMP signaling pathway was activated, which led to a greater proportion of yellow pigment in *Monascus*. In general, the experimental method of the high-strain yield brought by genetic engineering technology is more efficient and targeted; this is conducive to the sustainable utilization of resources to a certain extent.

We found that the ultrasonic-assisted extraction method is adequate for pigment extraction; under the action of ultrasonic waves and ethanol solvent, the pigment dissolved in ethanol. The optimal extraction process has been verified experimentally in terms of the concentration of ethanol, the liquid ratio, and the ultrasonic time for the extraction. A source of concern is the thermal effect of ultrasonic waves, which may affect the stability of the pigment; this will be our focus in subsequent studies.

There are many means of separating red yeast pigment. At present, the common methods include column chromatography, solvent extraction, and thin-layer chromatography. Loret et al. [48] extracted and characterized two new metabolites from *Monascus* cultures using thin-layer chromatography and semi-preparative liquid chromatography. Initially, the orange pigment was isolated successfully using thin-layer chromatography; however, the product obtained was still impure. There are still many unknown components of MPs, and separation methods need to be further explored.

One of the shortcomings of natural pigments is their poor stability, leading to quality degradation under the interference of factors such as temperature and light. Several studies have shown that *Monascus* culture at different temperatures resulted in different yields of MPs [49]. Wei et al. [50] investigated the use of sodium alginate and sodium caseinate, as well as calcium chloride, as crosslinkers; MPs were encapsulated through the ionic-gelation method, which had some positive effects on the stability. Yang et al. [51] improved the stability of the pigmentation by adding Flos sophorae immaturus to Hong Qu Huangjiu, resulting in an aromatic odor. The findings revealed that orange pigment had good thermal stability. Moreover, tea polyphenols reduced the degradation and exerted a protective effect on orange pigment; this was a great advantage for practical production in the food industry.

## 5. Conclusions

In this study, a strain RS7 with high-yield MPs was obtained through ARTP mutation breeding with laboratory-preserved *Monascus* R1 as the starting strain. Compared with the R1 strain, the color values of red, orange, and yellow pigment in the mutation strain RS7 increased by 75.1%, 66.7%, and 71.4%, respectively. HPLC detected a 69% reduction in citrinin content in the mutant strain RS7 compared to the original strain. Compared to traditional mutation breeding, ARTP mutagenesis has the advantages of a high mutation rate, a wide range of variation, and many beneficial mutations; these are important for improving efficiency and quality. Specifically, this process may be applied to obtain high-quality *Aspergillus erythrorhizus* mutant strains that are difficult to obtain with traditional breeding. MPs in the strains were obtained through ultrasonic extraction coupled with the response surface method to optimize the extraction conditions. The optimum extraction conditions were ethanol concentration of 81%, liquid material ratio of 39, and ultrasound time of 44 min. In this study, a strain with high orange pigment production was obtained; these findings provide scientific evidence that may be used to increase the production of orange pigment, which can greatly meet the color demand of food, textile, and cosmetic industries. These findings also lay the foundation for solving the current difficulties in the large-scale production of pigments using high-yielding strains and scaling up laboratory fermentations, which may be applied to industrial production.

## Data Availability

Data are contained within the article.
